# Vibrational density of states and thermodynamics at the nanoscale: the 3D-2D transition in gold nanostructures

**DOI:** 10.1038/srep39164

**Published:** 2016-12-16

**Authors:** R. Carles, P. Benzo, B. Pécassou, C. Bonafos

**Affiliations:** 1 CEMES-CNRS Université de Toulouse, rue Jeanne Marvig, BP 94347, 31055 Toulouse Cedex 4, France

## Abstract

Surface enhanced Raman scattering (SERS) is generally and widely used to enhance the vibrational fingerprint of molecules located at the vicinity of noble metal nanoparticles. In this work, SERS is originally used to enhance the own vibrational density of states (VDOS) of nude and isolated gold nanoparticles. This offers the opportunity of analyzing finite size effects on the lattice dynamics which remains unattainable with conventional techniques based on neutron or x-ray inelastic scattering. By reducing the size down to few nanometers, the role of surface atoms versus volume atoms become dominant, and the “text-book” 3D-2D transition on the dynamical behavior is experimentally emphasized. “Anomalies” that have been predicted by a large panel of simulations at the atomic scale, are really observed, like the enhancement of the VDOS at low frequencies or the occurrence of localized modes at frequencies beyond the cut-off in bulk. Consequences on the thermodynamic properties at the nanoscale, like the reduction of the Debye temperature or the excess of the specific heat, have been evaluated. Finally the high sensitivity of reminiscent bulk-like phonons on the arrangements at the atomic scale is used to access the morphology and internal disorder of the nanoparticles.

The size reduction of a solid towards the nanoscale has a strong impact on its lattice dynamics, namely on the occurrence of an acoustic gap and mode discretization. In fact, all the vibrational modes are affected and a distortion of the vibrational density of states (VDOS) is observed. As a consequence, size reduction generates “anomalies” – *i.e.* departures from the bulk behavior - on elastic and thermodynamic properties like lattice specific heat, thermal expansion, Debye and melting temperature, vibrational entropy…^1^,^2^,^3^,^4^,^5^,^6^. It also modifies the thermal and electrical conductivity because phonons mediate transport properties^7^,^8^,^9^. The smaller the size, the higher the role of surface atoms which are less coordinated than inner ones. The number of these surface atoms exceeds 50% for a NP of 2 nm in size, and they are known to greatly influence the stability, bond length, bulk modulus and obviously catalytic activity of nanostructures^10^. It has been shown that the VDOS is very sensitive to local morphologies because it realizes a dynamical signature of atomic arrangements and inter-atomic forces^4^,^11^,^12^. It is therefore of prime importance to get an understanding of correlations between lattice dynamics and atomic morphologies, and to assess experimentally the VDOS of nanostructures.

Noble metal clusters, nanoparticles (NPs), nanowires or thin films have attracted a particular attention owing to their plasmonics properties. Among noble metals, gold plays a peculiar role due to its relative stability and the occurrence of both interband electronic transitions and localized surface plasmon resonance (LSPR) in the visible optical range. Numerous studies have concerned their structural and optical properties (for a recent review, see Kossov *et al*.^13^), in particular their ability to induce localization of optical excitations^14^,^15^ and thus their application as surface-enhanced Raman scattering (SERS) substrates^16^.

As the primitive cell of gold contains a single atom, only acoustic-like vibrations are present. Most of the published works deal with their low frequency range (less than 1.5 THz, i.e. 50 cm^−1^) corresponding to linear dispersion of sound waves. In this range, finite size effects lead to the discretization of spatially confined modes referred as Lamb modes. In an elastically isotropic sphere, they are labelled by quantum numbers 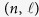, and their frequency varies as the inverse of the diameter. The low frequency fundamental (*n* = 1) modes may efficiently modulate the high polarizability of the confined electron gas and are easily detected in time-resolved pump-probe experiments or using Raman-Brillouin spectroscopy: for recent reviews on these two types of investigations, see Crut *et al*.^17^ and Saviot *et al*.^18^, respectively. These modes correspond to global deformations of the nanostructure and a continuous model of elasticity well accounts for their frequency dependence on size and shape modes^19^,^20^, or on intrinsic elastic anisotropy^21^.

However, the description of a NP as made up of a continuous medium with a given shape is too idealized for a deep understanding of its vibrational and thus thermodynamic properties. Only its description as a cluster of atoms with a specific organization allows to calculate all the vibrational frequencies and associated displacement vectors. In that way, a lot of works dealing with determinations of entire or local VDOS in noble metal NPs have been achieved through atomic simulations^5^,^11^,^12^,^19^,^20^,^22^,^23^,^24^,^25^,^26^,^27^,^28^,^29^. Let us resume the main results:

- All the models give a two-band shape of the VDOS reminiscent of transverse and longitudinal acoustical (TA and LA) modes in the bulk.
- The VDOS of nanoparticles is very sensitive to atomic arrangements (that may considerably differ between surface and inner atoms), twins and surface-disorder^11^,^12^,^25^.
- Some high frequency modes are observed beyond the cut-off of bulk VDOS and are generally attributed to a capillary pressure effect on the core atoms^26^,^28^.
-  An enhancement of the VDOS is observed at low frequencies and is generally attributed to a contribution of atoms located on surface or grain boundaries^22^,^25^.
- A question remains open concerning the frequency dependence in the Debye limit: does it evolves from quadratic to linear when reducing the size of the nanocrystals^22^,^23^,^27^?

Checking experimentally all these theoretical predictions is thus highly required. However exploring the whole VDOS of nanostructures is conceptually difficult because of their tiny volumes interacting with probes (neutrons or photons) and their instability under intense beams. Scarce results have been obtained on nanoclusters or nanocrystalline materials using nuclear resonant inelastic x-ray scattering (NRIXS) and inelastic neutron scattering (INS) techniques. The VDOS of nanocrystalline Ni and Fe^4^,^30^,^31^,^32^, Pt^2^, Pd^23^ or in epitaxial Fe layers^33^ have been extracted. In the case of noble metals, and in particular in Au, measurements are challenged by x-ray and neutron absorption and at that time, no experimental determination using either NRIXS or INS has been reported. However, plasmon-resonant Raman scattering has been recently revealed as an original way to access the VDOS of embedded Ag NPs or deposited Au colloids^28^,^29^. To circumvent, here also, the limits of a very low scattering volume and efficiency, spectrally and spatially localized surface plasmon resonance, optical amplification and dark-field geometry have to be simultaneously used^34^. Using atomic simulations, the effect of specific atomic arrangements has been checked by fitting the corresponding calculated VDOS with these experimental results^12^.

In this context, the first objective of this work was to use plasmon resonant Raman scattering to get an image of the VDOS of nude Au NPs in order to avoid several drawbacks of earlier attempts, as the presence of foreign bonds at interfaces with the matrix (embedded NPs) or ligands (capped colloids) that is known to affect the lattice dynamics of NPs^35^. A second objective was to explore the effect of size reduction, *i.e.* the concomitant increase of the surface over volume ratio, on thermodynamic properties. The third challenge was to get evidence of the “textbook 3D/2D transition” in the Debye regime^36^. By analyzing in details the low frequency part of the extracted experimental VDOS, this transition “bulk” to “surface” behavior has been observed, in agreement with previous theoretical expectations.

## Results

### Structural analysis

**Figure 1:**
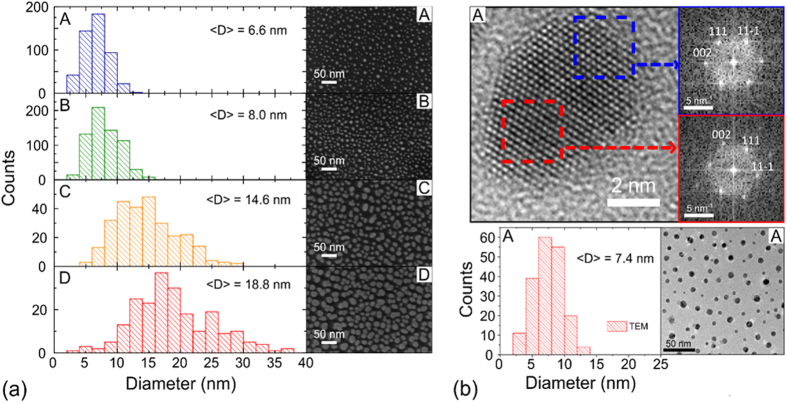
(**a**) Scanning Electron Micoscope images of the four samples (A to D) and their corresponding size histogram; the equivalent thickness of the Au deposit is 0.8, 1.6, 3.2 and 4.8 nm for samples A, B, C and D, respectively; (**b**) Transmission Electron Microscope (TEM) plan-view of sample A with its corresponding size histogram, and High-Resolution TEM view of an Au nanoparticle with the Fast Fourier Transform (FFT) corresponding to two different zones. This HRTEM image testifies a fcc crystalline ordering of pure gold; the presence of twins and facets can also be observed.

The images of four samples of Au NPs assemblies and the corresponding size distribution histograms are reported in Fig. 1(a). One notes that the NPs are crystalline, rather spherical with rather low size dispersion for samples A and B. Doubling the gold amount from A to B results in a slight increase of the mean diameter of the Au NPs (from 6.6 to 8.0 nm) and mostly in increasing their density. The NPs remain however isolated. The increase of the gold amount in samples C and D mainly results in a doubling of the mean size (14.6 and 18.8 nm). The shapes become elongated, strongly deviate from spheres with a size dispersion increase.

### Reflectance analysis

**Figure 2:**
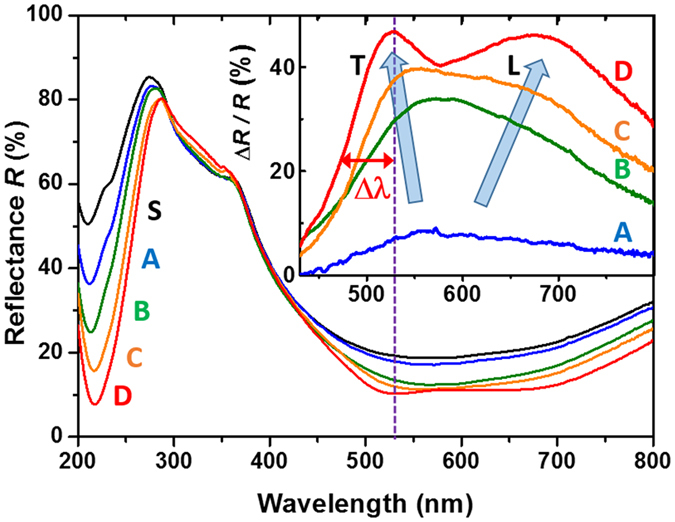
Reflectance spectra in the UV-visible range of the various samples with different equivalent thicknesses of the Au deposit: 0 (S), 0.8 (A), 1.6 (B), 3.2 (C) and 4.8 nm (D). In the inset the differential reflectance spectra are reported for the visible range; the two arrows indicate the transverse-longitudinal (T-L) splitting of the LSPR response; the dotted line indicates the laser wavelength used for the Raman measurements.

The optical reflectance spectra of the four samples are reported in Fig. 2 and compared with the signal coming from a zone without Au NPs (bare sample S). In this last spectrum, one clearly identifies two deep minima, one in the UV range (near 220 nm) and the other in the middle of the visible range (near 560 nm). They correspond to antireflective phenomena of the air/SiO_2_/Si bilayer when the dielectric thickness coincides with 3/4 or 1/4 of the light wavelength inside the dielectric, respectively. In between these minima, one identifies the singularities associated to direct optical transitions (at 295 and 365 nm) in the crystalline Si substrate.

The gold deposition is evidenced by its influence on these antireflective minima. On one hand, the increase of the gold equivalent thickness leads to a strong decrease (up to 80%) of the reflectance dip in the UV range. This simply reflects the absorption increase according to Lambert–Beer law, due to interband transitions in gold. On the other hand, a similar phenomenon is observed on the reflectance dip in the visible range, but with a more complex spectral signature. The differential reflectance spectra, defined as Δ*R/R* = 1 − *R/R*_0_ (*R*_0_ referring to sample S), are presented in the inset of Fig. 2. They show that the absorption attributed to the LSPR located near 560 nm for isolated and rather spherical NPs (sample A), progressively evolves towards a pronounced camel-back shape (sample D). This evolution is attributable to the transverse-longitudinal (L-T) splitting of the LSPR due to the elongation of the Au NPs. Similar behavior is expected when NPs collapse as early sign of the percolation threshold estimated at 53%. The surface coverage (51 ± 5%) for the 4.8 nm deposited thickness is indeed near this threshold. By simply ascribing the two humps near 530 and 675 nm to the T and L modes^3^, one found a shape ratio around 2.7, in reasonable agreement with the presence of oblate NPs in Fig. 1. Moreover collective effects due to NPs dipolar coupling between nearby NPs could be also referred to because they also lead to a redshift of the LSPR^15^. Important to note is the fact that the T mode of the LSPR remains at the vicinity of 530 nm for all the deposits ensuring their resonant excitation with the 531 nm laser line.

### Inelastic scattering by vibrational excitations

**Figure 3:**
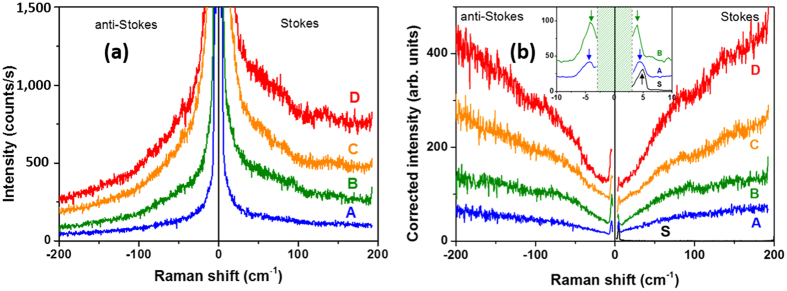
(**a**) Stokes and anti-Stokes Raman spectra of the different samples recorded at room temperature and under plasmon resonant conditions using the 531 nm Krypton laser line; (**b**) Corrected Raman spectra using equation (1). The low frequency range of three samples are presented in the inset with an enlarged frequency scale; for sample S, only the Stokes part showing the Brillouin mode of the Si substrate at 4.9 cm^−1^ is reported. The hatched area corresponds to the quasi elastic region which is masked by a mechanical filter. The equivalent thickness of the Au deposit is 0.8, 1.6, 3.2 and 4.8 nm for samples A, B, C and D, respectively.

Typical rough Raman spectra are displayed in Fig. 3(a) for both Stokes and anti-Stokes ranges. Using specific dark-field geometry and filtering^34^,^37^, the spectra have been recorded with frequency shifts down to few cm^−1^. Some very weak structures are visible around 80 and 140 cm^−1^ superimposed on a strong quasi-elastic scattering tail which intensity increases rapidly with the Au equivalent thickness (from A to D). In order to verify that the collected signal comes from Raman processes, and not luminescence ones, the rough signal *I*^exp^ has been corrected by the Bose statistical population factor 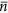 and by the spectral response *f* of the whole experimental set-up^34^. One thus obtains a “corrected” intensity:


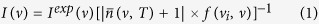


where *ν* is the Raman frequency shift (*ν* > 0 for Stokes, and *ν* < 0 for antiStokes), and *ν*_*i*_ the laser frequency.

One clearly observes in Fig. 3(b) that the corrected spectra are perfectly symmetrical with regard to the Rayleigh line testifying that the recorded signal obeys the thermodynamic balance equation and thus originates in thermalized Raman processes. As already discussed in details recently^28^,^29^,^34^, the inelastic scattering contributions are due to either atomic or electronic excitations. Among the contributions of atomic motions, Lamb modes imply each NP as a whole and they are well described within the elastic approximation of a continuous medium^17^. They give the most intense contribution in resonant plasmon inelastic scattering because of their spatial phase matching with the dipolar plasmon. For an isolated sphere the lowest frequency and highest intensity Raman response is due to the fundamental quadrupolar mode 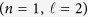
38. According to the linear dispersion of acoustic modes, its frequency varies as the inverse of the diameter *D*. Assuming an icosahedron ordering and neglecting intrinsic anisotropy, the following expression has been proposed theoretically and well verified experimentally for Au NPs^21^: 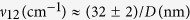. In the inset of Fig. 3(b), one clearly distinguishes this mode for the lowest Au NP sizes, at 4.4 ± 0.2 and 3.4 ± 0.2 cm^−1^, for sample A and B, respectively. One thus obtains *D* ≈ 7.3 ± 0.8 and 9.4 ± 1.2 nm, in rather good agreement with electron microscopy observations (see Table 1). For samples C and D, this mode (expected near 2.4 and 2 cm^−1^, respectively) is hidden by the filter cut-off near the Rayleigh line, at 3 cm^−1^.

### Extraction of the VDOS

**Figure 4:**
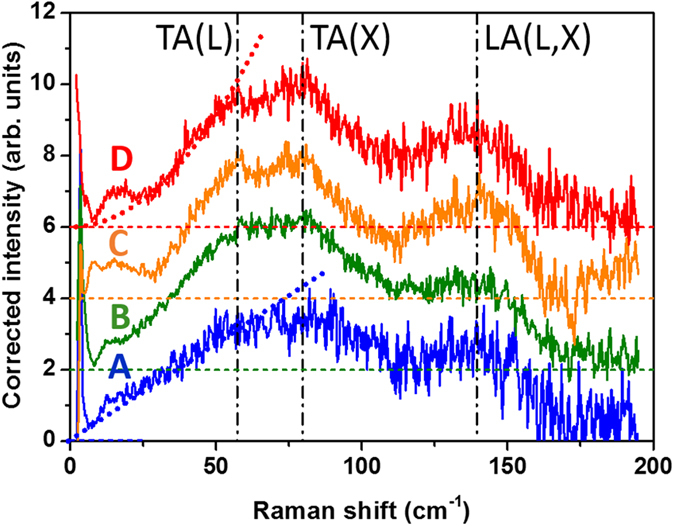
Raman spectra corresponding to the lattice vibration contribution for the different samples (A to D). The integrated intensities have been normalized and the spectra arbitrary up-shifted for an easier comparison. The equivalent thickness of the Au deposit is 0.8, 1.6, 3.2 and 4.8 nm for samples A, B, C and D, respectively.

**Figure 5:**
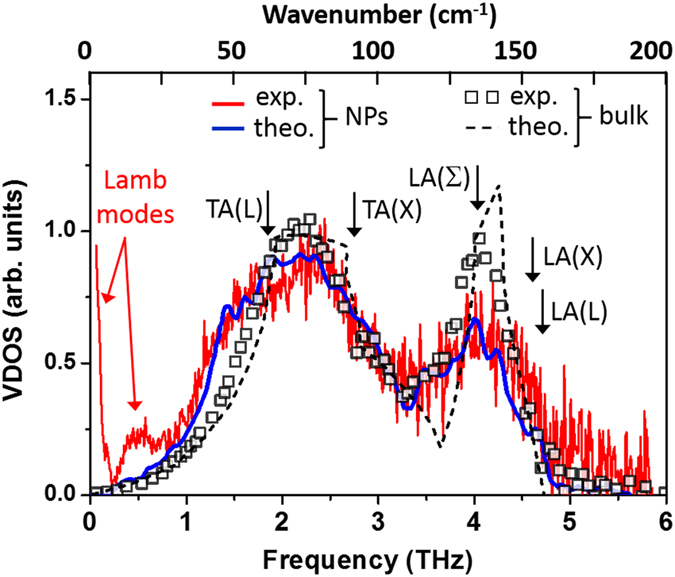
VDOS in bulk gold (empty squares: extracted from INS experiments^41^, dotted lines: calculated using a fourth-neighbor general force model^39^, and in gold nanoparticles (in blue: simulations for an icosahedral Au NP^12^, in red: experiment from present Raman data on sample D corresponding to an Au equivalent thickness of 4.8 nm.

We now focus on the numerous other vibrations of atoms that make up each NP. They only give a hardly noticeable signal that superimposes the electronic response below 200 cm^−1^ in Fig. 3(b). For each deposit, we have taken advantage of plasmon resonance and optical amplification, but also of x-y scanning in order to record hundreds of spectra. The corrected and averaged spectra are reported in Fig. 4; the substrate contribution has been subtracted from records on a zone without NPs, and the electronic contribution was also subtracted using a linear fit (see Supplementary information Fig. S1). All the spectra reported in Fig. 5 now clearly show evidence of Van Hove singularities of the Au VDOS near points of high symmetry of the Brillouin zone, like L or X. These reminiscences of a reciprocal space symmetry testify the crystalline ordering of all the samples, in agreement with HRTEM observations (Fig. 1). In Fig. 4, the dash-dotted lines correspond to the frequencies of transverse (TA) or longitudinal acoustical (LA) frequencies measured by INS in bulk gold^39^. At low frequency, below 20 cm^−1^ some weak features are due to overtones of Lamb modes and they will be discarded in the following discussion.

When the mean size of the Au NPs increases, from sample A to D, one observes that (*i*) the structures become better defined, (*ii*) the high frequency band (LA band) is less flat, and (*iii*) the frequency dependence of the low energy tail of the TA band evolves from linear to quadratic (see dotted lines in Fig. 4). How a corrected Raman spectrum can be fruitfully directly compared with a theoretical determination of the VDOS has been recently presented on assemblies of gold colloidal nanocrystals^28^ and will not be discussed in details here. Until the pioneering work of Shuker and Gamon^40^, it has been shown that, owing to the lack of translational invariance in a material, the corrected intensity of Raman scattering by vibrations should be directly linked to the VDOS, *g*(*ν*), following:


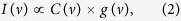


The “coupling coefficient” *C*(*ν*) is expected to be weakly dependent on the vibrational frequency out of very selective electronic transitions, and *I*(*ν*) has been shown to give a very accurate image of the VDOS^28^,^29^,^34^.

Let us focus on the result concerning sample D where the signal is the most clearly defined. It is reported in Fig. 5 (red curve) and compared with the neutron-weighted VDOS in bulk Au (empty squares) deduced from INS^41^, the VDOS of bulk Au (dotted line) calculated using a fourth-neighbor general force model^39^ and the theoretical VDOS (continuous blue line) of an icosahedral Au NP containing 2057 atoms^12^. This last determination has been obtained from atomistic calculations based on a semi-empirical tight-binding many-body Gupta potential^42^ in which a 1.2 scaling factor has been applied to the frequency scale to fit the phonon frequencies in bulk Au, as already noted^12^,^20^,^28^,^42^.

For an easier comparison between NPs with different ratio of surface atoms versus volume ones (*ie.* with different sizes), all the integrated areas in Figs 4 and 5 have been scaled to obtain the same value 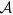. This value normally equals the number *N*_*m*_ of vibrational modes of the NP composed with *N*_*at*_ atoms, excluding global translations and rotations:


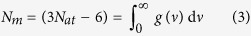


In Fig. 5, the two determinations of the VDOS in bulk Au are quite similar over the whole frequency range. This is easily understandable if one notes that the force constants of both models^12^,^41^ have been adjusted to account for the same phonon frequencies measured by INS^39^. All these spectra display a parabolic shape at low frequencies as expected from a 3D Debye-like behavior. The TA-like band is wide in contrast to the well-defined LA band near 4 GHz (135 cm^−1^) ending with a sharp cut-off at the LA(L) frequency at 4.70 GHz (158 cm^−1^)^39^. One also notes that in all the spectra reported in Figs 4 and 5, the area of the TA band is twice that of the LA one, as expected from the twofold degeneracy of TA-like modes along a large part of the Brillouin zone. This confirms that in equation (2) the coupling factor *C*(*ν*) is not too frequency- (or band-) dependent as this area ratio is not affected when going from the bulk to NPs (Fig. 5) or when changing the size (Fig. 4).

Focusing at first on the Debye regime for frequencies *ν* ≲ 1.2 THz, one can fit the various VDOS reported in Fig. 5 with the theoretical Debye *ν*^2^-dependence^36^:


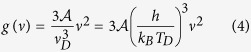


and thus deduce in each case an effective Debye temperature *T*_*D*_. From the data reported in Fig. 5, one gets for bulk gold *T*_*D*_ = 167 ± 5 K, in good agreement with the well admitted value of 170 K^36^. One clearly observes in Fig. 5 that the VDOS in NPs exceeds that of the bulk at low frequencies, thus leading to a lowering of the corresponding Debye temperature, according to equation (4). Using the experimental data from sample D, one gets *T*_*D*_ = 138 ± 10 K, in perfect agreement with simulations^12^ (*T*_*D*_ = 138 ± 10 K). This lowering of *T*_*D*_ versus the size reduction has been already observed experimentally in Au thin films^1^, in nanocrystalline Fe^43^ or colloidal Au NPs^28^. Theoretical models based on atomistic simulations and on elasticity models predict a strong decrease of *T*_*D*_ for free nanocrystals because of the surface-stress effect^11^,^44^. This tendency is clearly confirmed in this work on nude and isolated gold nanostructures, thus ruling out previous hypothesis on the role of oxide layers^27^ or grains. It is very important because the Debye temperature decrease can be correlated to a concomitant decrease of thermal stability or electrical conductivity which are major parameters in the upcoming technologies using gold nanostructures.

## Discussion

By examining more globally the VDOS in Au NPs reported in Fig. 5, one notes that the experimental spectrum extracted from Raman data is in remarkable agreement with atomistic calculations^12^ over the whole frequency range (excluding the quantized modes at very low frequencies). First, that confirms the ability of our approach based on inelastic scattering by visible photons to access accurate information on the whole vibrational properties of disordered systems^40^ or nanostructures^28^,^29^. Second, the best fit is obtained for the icosahedral (ICO) morphology for the Au NPs. Calculations performed using the same interatomic parameters but with other morphologies (not reported here, see ref. 12), either face-centered cubic (FCC) or decahedral (DEC), give a poor agreement with experiment; in particular, the smearing of the LA band, similar to what is observed when going from the bulk to a reduced size (Fig. 5), is not at all well accounted for.

A crude description of the flattening of peaks in the VDOS could be viewed as a simple reduction of the correlation volume of all the vibrational modes^32^,^40^. Indeed, due to confinement within each NP of size *D*, plane waves in the bulk are changed into wave packets in this NP: the resulting momentum spreading Δ*k* ~ 2*π/D* would induce a frequency broadening Δ*ω* = *v*_*g*_Δ*k* of each mode according to its specific group velocity *v*_*g*_. However the modes that give the higher contribution to the VDOS belong to high symmetry points of the Brillouin zone where *v*_*g*_ is known to vanish. The confinement or disorder effect is not able to account alone for the strong distortion of the TA and LA bands. It also fails to explain specific observations (Fig. 5), like: (i) the occurrence of some modes at higher frequencies (around 5.1 THz) than the bulk cut-off (4.7 THz)^39^, (ii) the strong and asymmetric broadening of the LA band (around 4 THz) and (iii) the enhancement of the VDOS on the low frequency tail of the TA band (between 0.8 and 1.6 THz). The same observations have been already noted in some nanocrystalline monoatomic metals (Fe, Pt, Pd, Fe, Ni) using INS or NRIXS^2^,^4^,^23^,^27^,^30^,^31^,^32^ or very recently in Au and Ag using Raman scattering^28^,^29^. These features are in good agreement with atomic simulations in various metal NPs^5^,^11^,^12^,^20^,^22^,^25^,^26^,^45^. This work presents experimental proof that such “anomalies” are intrinsic phenomena also present in isolated and nude noble metal nanoparticles, excluding multigrain effects^11^, the presence of surface oxides^27^,^32^, capping molecules^28^ or surrounding matrix^29^.

In Fig. 1(b), the HRTEM image of an isolated NP shows clear evidence of the presence of twins in the present samples. The role of twins has been generally neglected in atomic simulations of lattice dynamics of nanoparticles and obviously in all the models based on a continuous elastic medium. However twinning in small particles appears as a common phenomenon because the energy gained by the removal of twin boundaries cannot compensate the loss in surface energy^44^. A variety of polyhedral models have been proposed to explain results of electron diffraction and imaging^45^,^46^,^47^,^48^. In particular, resulting stable decahedral (DEC) and icosahedral (ICO) structures have been successfully employed to account both for stability criteria and for interpreting HRTEM images. These structural models can be approximately described as arising from a set of fcc tetrahedral segments joined together in specific twin relationships. These arrangements induce some frustrations among which the resulting five-fold symmetry has been largely debated: the presence of angular deficits give rise to internal distortions^47^. These structural considerations will have obviously their signatures in the lattice dynamics of NPs. The presence of multiply twinned Au NPs has been shown to erase the degeneracy lifting of the quadrupolar mode 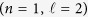 due to intrinsic elastic anisotropy^21^. Similar effects should indeed concern all the vibrational modes and a high sensitivity of the VDOS to morphology at the atomic scale has been predicted by several atomistic simulations^5^,^11^,^12^,^29^.

In that sense, the occurrence of modes at higher frequencies than in the bulk (i) has been theoretically attributed to a global shrinking of the NPs^22^ or a capillary pressure on core atoms^26^ which causes an upshift of the longitudinal modes. More precisely these modes, localized on inner atoms, are particularly affected by the twinning-induced distortions. Indeed, the presence of larger strains and twining in the ICO morphology of Ag and Au NPs has been suggested^11^.

The broadening of the LA band (ii) which intensity maximum is lower than the TA band in a NP also appears in calculations as a signature of the ICO or DEC morphology^11^, and even more specifically of only the ICO one^12^. The perfect agreement of our experimental determination of the VDOS with this last theoretical prediction comforts the hypothesis of a predominance of this morphology in noble metal NPs. This hypothesis is also reinforced by energetic considerations that predict the highest stability for a NP with icosahedral multiply-twinned morphology^45^. The LA broadening can be understood as resulting not only from strain and confinement effects, but mainly from symmetry breakdown at twin boundaries and at surfaces. The highest intensity features of the VDOS are linked to Van Hove singularities at high symmetry points (X, L, K) at the Brillouin zone edge. The corresponding wavelength of the vibrational modes are of the order of the interatomic spacing and consequently highly perturbed by geometry changes at the atomic scale. A similar argument has been recently invoked to account of optical properties in silicon NPs through the broadening of the distribution of electron wave functions in the k-space (Γ-X coupling)^49^.

The presence of excess modes at low frequencies has been already observed in INS in nanocrystalline Fe deposits^31^ and theoretically associated in Ag NPs to contributions of the surface atoms^22^ which have a coordination number lower than the inner value of 12. By examining the effective number of atoms participating to a given frequency mode, it has been shown that the more localized the vibrations at the surface, the higher the enhancement of the VDOS at low frequencies^11^. In order to check this contribution we focus on the specific frequency range 25–50 cm^−1^, located in between the upper contribution of Lamb modes and the curvature of the TA branches. In this range the Debye approximation is thus fulfilled because all the dispersion curves are linear^39^. On one hand, one notes in Fig. 5 that only this range exhibits a higher population of modes than in the bulk. On another hand, one observes that the corresponding frequency dependence is strongly modified, from quadratic to linear, when reducing the average size of the NPs (from sample D to A in Fig. 4).

**Figure 6:**
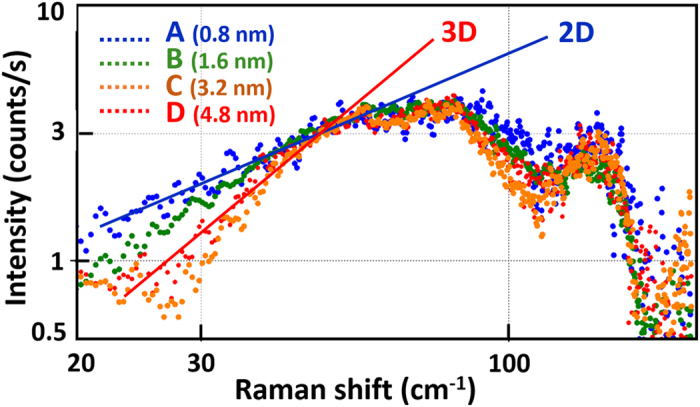
Log-log plot of the VDOS extracted from the Raman data of 4 samples (A to D). The straight lines refer to the Debye approximation (slopes values equal 1 or 2 for dimensionality 2 or 3, respectively).

To exemplify these phenomena, the experimental VDOS are plotted in Fig. 6 using a log-log scale. Whereas the high frequency part of the different spectra are rather unchanged, one clearly observes the transition from a 3D behavior for sample D to a 2D behavior for sample A: the slope changes from *n* ≈ 2 (quadratic dependence) to *n* ≈ 1 (linear dependence). One thus confirms the prominent role of surface modes in the low frequency range for nanometer-sized nanoparticles. This transition has been predicted by some atomistic simulations but remains controversial in experiments^4^,^22^,^23^,^24^,^25^,^31^,^43^. The power *n* in the dependence of *g*(*ν*) ∝ *v*^*n*^ at low frequencies is still the subject of debate mainly because the analysis performed on different assemblies of NPs suffer from the uncertainties on data corrections and heterogeneities of the samples. Very recently the 2D–3D transition has been clearly observed in ultrathin FeO films but it concerns nonmetallic systems containing also optical modes and whose acoustic modes are only confined in one direction^50^.

**Figure 7:**
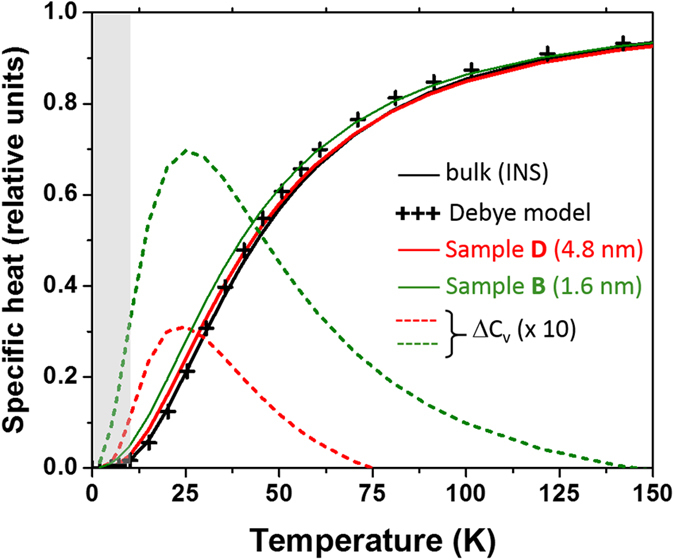
Normalized specific heat deduced from the Raman data on Au nanoparticles (samples B and D). They are compared with the specific heat of bulk gold using the density of states deduced from neutron data^39^ and from the Debye model using equation (4) with *T*_*D*_ = 167 K (crosses). The excess specific heat for both samples are also reported (dotted lines) with a multiplication factor of 10. The shaded area corresponds to quantized modes.

The specific heat of a solid *C*_*v*_(*T*) is essentially dominated by the lattice contribution at high temperature *T* because the electronic contribution becomes negligible^36^. When *T* is higher than the Debye temperature *T*_*D*_, *C*_*v*_(*T*) tends toward the classical limit *C*_*v*,∞_ = 3*N*_*m*_*k*_*B*_. In order to compare the thermal behavior of samples with different sizes, we define a normalized specific heat *C*_*v*_(*T*), using equation (3), as follows:
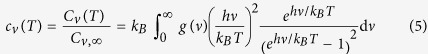


In the density of states *g*(*ν*) deduced from Raman data, the selective resonant contribution of the few Lamb modes has been arbitrary subtracted (see dotted lines for samples A and D in Fig. 4). This correction affects the VDOS in its discrete part at the vicinity of the acoustical frequency gap (*ν*_*gap*_). Our calculation are thus obviously valid in the high temperature range (*T* > 10 *K*) where this quantized regime is blurred by the thermal effect (*k*_*B*_*T* ≳ *hν*_*gap*_). The calculated specific heat *c*_*v*_(*T*) corresponding to samples B and D is reported in Fig. 7. One observes that the Debye approximation is perfectly verified for bulk gold at low temperature (*T* ≲ 30 K) whereas an excess specific heat is noted for Au NPs. This excess can be accounted for by introducing an “effective” Debye temperature, lower than in the bulk, as discussed previously.

In Fig. 7 the excess is deduced by subtracting the bulk contribution, in order to clearly show that it essentially affects the behavior at low temperature (*T* ≪ *T*_*D*_) because of the prominent role of surface contribution. As a matter of fact, the excess has more than doubled (3% to 7%) when the mean size is reduced from sample D to B. The excess specific heat has been already examined when going from bulk to nanocrystalline copper, either theoretically in clusters^26^ or experimentally^6^. In this last and very recent work, the excess has been shown to reach a maximum of 5% near 50 K, in good agreement with the present observation.

This work has thus experimentally confirmed a lot of theoretical phenomena predicted during the least decades, like the distortion of the VDOS induced by size reduction, internal disorder and strain, the 2D–3D transition when surface effects become prominent, and the resulting “anomalies” in thermodynamic properties.

## Methods

### Elaboration of the samples

The Au NPs were grown on specific SiO_2_(90 nm)/Si substrates by dc magnetron sputtering of a pure gold target in a Plassys ultra-high-vacuum device. The metallic layer was deposited at 600 °C in order to ensure the formation of NPs. The current was chosen to achieve a deposition rate of 0.027 nm/s. The amount of deposited gold was calibrated by the mass-equivalent thickness (Table 1). Four samples, labelled from A to D, have been selected to get separated NPs with different sizes and shapes. On each sample a zone without deposit is preserved and referred as S in all the presented results.

### Structural analysis of the samples

A GEMINI field emission column integrated in a Zeiss CrossBeam 1540 was used for Scanning Electron Microscopy (SEM) imaging. The crystalline nature of the NPs has been demonstrated using high-resolution transmission electron microscopy (HRTEM) in a FEI Tecnai microscope equipped with a field emission gun and a spherical aberration corrector and operating at 200 keV.

### Optical characterization of the samples

The reflectance of the samples has been recorded in quasi-normal incidence using a Varian Cary 5000 UV-visible spectrometer. The Raman spectra were recorded at room temperature in air, using a T64000 Horiba Jobin Yvon triple spectrometer. To record the scattered signal down to few cm^−1^ from the Rayleigh line, the standard backscattering geometry is replaced by a two-objective system. The incoming laser beam is focused under an oblique incidence on the sample through a first objective (12 mm focal length); the scattered light is collected in the normal direction by a long working distance microscope objective (numerical aperture: 0.55, magnification: 40). Moreover, a specific optical and electronic filter is inserted into the spectrometer to limit the spectral window (See ref. 34 for details). The Raman spectra of the samples have been recorded under plasmon resonant and antireflective conditions using the 531 nm line of a Krypton laser as excitation (see dashed line in Fig. 2).

## Additional Information

**How to cite this article:** Carles, R. *et al*. Vibrational density of states and thermodynamics at the nanoscale: the 3D-2D transition in gold nanostructures. *Sci. Rep.* **6**, 39164; doi: 10.1038/srep39164 (2016).

**Publisher's note:** Springer Nature remains neutral with regard to jurisdictional claims in published maps and institutional affiliations.

## Supplementary Material

Supplementary Information

## Figures and Tables

**Table 1:** Structural characteristics of the samples.

Sample	Equivalent thickness (nm)	Diameter (nm) from MEB	Diameter (nm) from Raman	Covered area (%)
S	0	—	—	0
A	0.8	6.6 ± 1.9	7.3 ± 0.8	16 ± 2
B	1.6	8.0 ± 2.4	9.4 ± 1.2	23 ± 3
C	3.2	14.6 ± 4.9	>10	42 ± 4
D	4.8	18.8 ± 6.3	>10	51 ± 5
