# Supplementary material for: Vibrational density of states and thermodynamics at the nanoscale: the 3D-2D transition in gold nanostructures
Source: Sci Rep. 2016 Dec 16;6:39164. doi: 10.1038/srep39164 (PMC5159851; doi:10.1038/srep39164)
Supplement: Supplementary Information [file srep39164-s1.pdf]

SUPPLEMENTARY INFORMATION

Vibrational density of states and thermodynamics at the nanoscale: the 3D-2D transition in gold nanostructures

R. Carles*, P. Benzo, B. Pécassou and C. Bonafos

CEMES-CNRS Université de Toulouse, rue Jeanne Marvig,

BP 94347, 31055 Toulouse Cedex 4, France

Raman scattering by electron-hole excitations in conducting materials consists in a huge signal which frequency dependence is rather linear at low frequency and slowly decreasing at high frequency. This contribution, first revealed on rough Ag surfaces [S1] has been analyzed experimentally [S2, S3]. Very recently it has been accounted for theoretically [S3] using an approach developed for Raman scattering in dirty metals [S4]. Let us briefly record the main salient features of this theoretical approach. An elementary electronic Raman scattering process in a metal consists in the excitation of an electron from below to above the Fermi level. In the particular case of bulk noble metals, electronic Raman scattering is expected to be negligible because such intraband excitation is forbidden due to momentum conservation rules. In a finite size system or a rough surface, this barrier is lifted and a continuum of electronic excitations become Raman active. The corresponding normalized and temperature-corrected Raman intensity for both Stokes and anti-Stokes processes, can be written as [S3-S5]:

$$I_{e-h} \propto \frac{2\pi\,|\nu|\tau_s}{1+\ (2\pi\nu\tau_s)^2} \qquad \text{(S1)}$$

where $\tau_s = D^*/v_F$ is an "effective" collision time, v_F the Fermi velocity and D^* the corresponding electronic mean free path between collisions. To ensure that the background of the spectra reported in Fig. 3(b) are really due to such electronic contribution, we have analyzed it over an extended frequency range. An example of this contribution for the thickest sample D

is reported in Fig. S1 and has been obtained as follows. First, the signal recorded within the same experimental conditions on sample S (originating in the SiO_2/Si substrate) has been subtracted. Second, the resulting spectrum has been corrected for the Bose factor $\overline{n}$ and set-up response, using equation (1).

The final spectrum reported in Fig. S1 has been fitted with the theoretical expression given by equation (S1), using τ_s as the only adjustable parameter. The theory is in remarkable agreement with the experiment, apart for two bands (marked with blue arrows in Fig. S1). They correspond to SERS by C-C bonds of some contaminating organic molecules deposited on the sample. The maximum of the electronic Raman spectrum is observed for a shift around 1700 cm^{-1}. From this value one gets $\tau_s \approx 3.1$ fs and $D^* \approx 4.4$ nm which can be considered as a reasonable value in Au NPs presenting a wide size distribution at the nanoscale (see Table 1), with shape irregularities and suspected twins. Indeed it has been shown that D^* gives a measure of the grain size or the distance characteristic of internal defects or surface roughness, all of them limiting the electronic mean free path [S3]. This value of τ_s would correspond to a broadening of the plasmon resonance, $\Gamma_s = \hbar/\tau_s \approx 210$ meV. One can independently estimate this broadening from the half width Δλ of the transverse plasmon peak located at $\lambda_p = 530$ nm (i.e. $E_p = 2.40$ eV) for sample D (see inset in Fig. 2). With $\Delta\lambda \approx 50$ nm, one indeed obtains a good support for our interpretation as we gets: $\Gamma = E_p \times (\Delta\lambda/\lambda_p) \approx 230$ meV. It is important to note that our interpretation of the so-called "background" in SERS spectra discards here the role of photoluminescence processes. The role of ligands in colloidal solutions or defects in the surrounding matrix is irrelevant in the present case. However, two-photon-induced photoluminescence has been observed in gold nanorods corresponding to interband transitions at the L or X points of the Brillouin zone, located around 550 or 650 nm respectively [S6]. The corresponding Raman shifts would be around 650 or 2450 cm^{-1} but no structure is observed in Fig. S1.

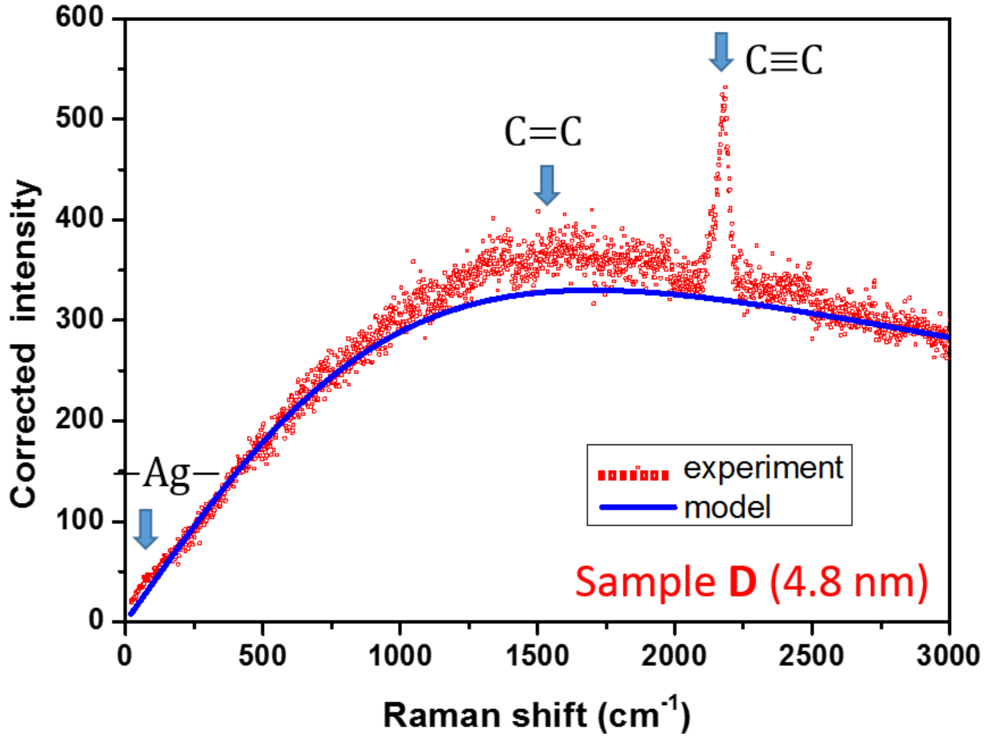

Figure S1: Electronic contribution to Raman scattering in Au NPs. The corrected Stokes Raman spectrum (dots) of sample D is recorded over an extended frequency range. The continuous line corresponds to a theoretical fit. The arrows indicate molecular SERS signals associated to carbon contamination (C=C stretching around 1500 cm^{-1} and alkyne C≡C stretching at 2100 cm^{-1}) or to Ag vibrations below 200 cm^{-1}.

[S1] Akemann, W., Otto, A. & Schober, H. R. Raman scattering by bulk phonons in microcrystalline silver and copper via electronic surface excitations. *Phys. Rev. Lett.* **79**, 5050 (1997).

[S2] Portales, H., Duval, E., Saviot, L., Fujii, M., Sumitomo, M. & Hayashi, S. Raman scattering by electron-hole excitations in silver nanocrystals. *Phys. Rev. B* **63**, 233402 (2001).

[S3] Carles, R. *et al*. Plasmon-resonant Raman spectroscopy in metallic nanoparticles: Surface-enhanced scattering by electronic excitations. *Phys. Rev. B* **92**, 174302 (2015).

[S4] Zawadowski, A. & Cardona, M. Theory of Raman scattering on normal metals with impurities. *Phys. Rev. B* **42**, 10732-10734 (1990).

[S5] Devereaux, T. P. & Hackl, R. Inelastic light scattering from correlated electrons. *Rev. Mod. Phys.* **79,** 175 (2007).

[S6] Imura, K., Nagahara, T. & Okamoto, H. Near-field two-photon-induced photoluminescence from single gold nanorods and imaging of plasmon modes. *J. Phys. Chem. B* **109**, 13214-13220 (2005).
